# Ancillary Diagnostic Utility of Glycoprotein Nonmetastatic Melanoma Protein B (GPNMB) in Diagnostically Challenging Hepatic Angiomyolipoma

**DOI:** 10.7759/cureus.110598

**Published:** 2026-06-10

**Authors:** Suyeon Kim, M. Zaki Karzoun, Yan Zhou, Minghao Zhong, Paari Murugan, Robben Schat, Jacob Ankeny, Byoung U Park

**Affiliations:** 1 College of Medicine, University of Ulsan, Seoul, KOR; 2 Department of Laboratory Medicine and Pathology, University of Minnesota, Minneapolis, USA; 3 Department of Laboratory Medicine and Pathology, University of Minnesota, MInneapolis, USA; 4 Department of Radiology, University of Minnesota, Minneapolis, USA; 5 Department of Surgery, University of Minnesota, Minneapolis, USA

**Keywords:** angiomyolipoma, diagnostic pathology, gpnmb, hepatic angiomyolipoma, liver tumor

## Abstract

Hepatic angiomyolipoma (HAML) is a rare mesenchymal neoplasm within the perivascular epithelioid cell tumor (PEComa) family that may pose significant diagnostic challenges because of its variable radiologic, histologic, and immunophenotypic features. HAML demonstrates a broad morphologic spectrum, including component-predominant and other uncommon histologic variants, which may obscure the classic triphasic morphology, particularly in limited biopsy specimens. Recently, glycoprotein nonmetastatic melanoma protein B (GPNMB) has emerged as a sensitive immunohistochemical marker for PEComa-family tumors.

A 39-year-old man with a history of Hodgkin lymphoma presented with intermittent abdominal pain and an enlarging abdominal mass. Imaging demonstrated a hypervascular fat-containing lesion arising from the left hepatic lobe. Core biopsy revealed a mesenchymal neoplasm composed of mature adipose tissue, epithelioid cells, and thick-walled blood vessels without significant atypia or mitotic activity. Immunohistochemical studies demonstrated focal, patchy positivity for HMB45 and Melan-A; diffuse positivity for GPNMB and smooth muscle actin (SMA); and negativity for HepPar-1, glypican-3, CAM5.2, CK7, S100, and desmin. The morphologic and immunophenotypic findings supported the diagnosis of HAML. The patient subsequently underwent left lateral segmentectomy, and surgical pathology confirmed hepatic angiomyolipoma with negative margins. The postoperative course was uncomplicated.

HAML remains a diagnostically challenging hepatic neoplasm because of its broad morphologic spectrum and variable radiologic and immunophenotypic features. Component-predominant and other uncommon variants may complicate recognition of the classic triphasic morphology, particularly in limited biopsy specimens. This case highlights the potential diagnostic utility of GPNMB as an ancillary immunohistochemical marker in challenging HAMLs that demonstrate limited or focal expression of conventional melanocytic markers, such as HMB45 and Melan-A.

## Introduction

Angiomyolipoma (AML) is a mesenchymal neoplasm with predominantly benign behavior, composed of a mixture of abnormal blood vessels, smooth muscle cells, and adipose (fat) tissue [1]. AML belongs to the perivascular epithelioid cell tumor (PEComa) family, which also includes lymphangioleiomyomatosis (LAM), clear cell "sugar" tumor of the lung, and PEComa-not otherwise specified (NOS). PEComa is a broad family of rare mesenchymal neoplasms characterized by distinctive perivascular epithelioid cells with features of melanocytic and smooth muscle differentiation [2].

While AMLs primarily occur in the kidney, they can be found infrequently in various extrarenal locations throughout the body. The liver represents one of the extrarenal sites where AMLs can be identified. Other reported extrarenal locations include bone, colon, heart, lung, parotid gland, skin, spermatic cord, gynecologic regions, and retroperitoneum [3].

The diagnosis of hepatic angiomyolipoma remains challenging due to its imaging characteristics mimicking hepatocellular carcinoma and other hyper-vascular liver tumors. The imaging appearance of hepatic angiomyolipoma (HAML) varies with the relative proportions of blood vessels, smooth muscle, and adipose tissue. A paucity of adipose tissue renders radiologic diagnosis particularly challenging [1,4].

Given these challenges, definitive diagnosis relies on histopathologic examination with immunohistochemistry. AMLs belong to the PEComa family, which is defined by the co-expression of melanocytic and smooth muscle markers. The use of key melanocytic and smooth muscle markers such as HMB-45, Melan-A, and smooth muscle actin (SMA) is essential for the diagnosis of HAML [1].

However, these traditional markers can be focal or equivocal in some cases, posing diagnostic challenges. Recently, glycoprotein nonmetastatic melanoma protein B (GPNMB) has emerged as a novel, highly sensitive immunohistochemical marker for the PEComa family, often surpassing the sensitivity of traditional melanocytic markers. Therefore, GPNMB may serve as a valuable diagnostic tool for HAML, particularly in the context of focal expression of conventional melanocytic markers [5,6].

In this report, we present a diagnostically challenging case of HAML with limited expression of conventional melanocytic markers, in which GPNMB provided important supportive diagnostic evidence.

## Case presentation

A 39-year-old man with a history of Hodgkin lymphoma treated with chemotherapy and radiation therapy during adolescence, hypertension, hyperlipidemia, and prior intestinal volvulus requiring bowel resection presented with an enlarging abdominal wall mass and intermittent right lower abdominal/flank pain. He first noticed the abdominal wall mass several months before presentation and reported progressive enlargement without associated tenderness. He also described intermittent sharp right lower abdominal and flank pain exacerbated by prolonged sitting and partially relieved by ibuprofen. The patient worked in an assembly position involving occasional heavy lifting. No documented exposure to industrial chemicals, radiation, or other relevant occupational hazards was identified. Physical examination revealed a firm, mobile, non-tender subcutaneous mass within the right upper abdominal wall corresponding to a clinically suspected lipoma. The abdomen was otherwise soft and non-tender without palpable intra-abdominal masses, hepatomegaly, or signs of chronic liver disease.

Laboratory evaluation demonstrated hypercholesterolemia and a hemoglobin A1c of 6.1%, consistent with prediabetes. Liver function tests were within normal limits (aspartate aminotransferase 20 U/L, alanine aminotransferase 29 U/L, alkaline phosphatase 77 U/L, total bilirubin 0.4 mg/dL). Serum alpha-fetoprotein (3.1 ng/mL) and carbohydrate antigen 19-9 (8 U/mL) were not elevated (Table 1).

**Table 1:** Relevant laboratory findings at presentation

Laboratory test	Result	Reference range
Hemoglobin A1c	6.10%	<6.4 %
Total cholesterol	266 mg/dL	<=200 mg/dL
Alpha-fetoprotein (AFP)	3.1 ng/mL	<=8.3 ng/mL
Carbohydrate antigen 19-9 (CA 19-9)	8 U/mL	<=35 U/mL
Aspartate aminotransferase (AST)	20 U/L	0 - 45 U/L
Alanine aminotransferase (ALT)	29 U/L	0 - 70 U/L
Alkaline phosphatase	77 U/L	40 - 150 U/L
Total bilirubin	0.4 mg/dL	<=1.2 mg/dL

Initial radiologic work-up with abdominal ultrasound demonstrated a 7.5 × 6.2 × 6.0 cm heterogeneous, lobulated, hyperechoic, vascular mass located in the upper abdomen between the left hepatic lobe and the pancreatic head (Figure 1).

**Figure 1:**
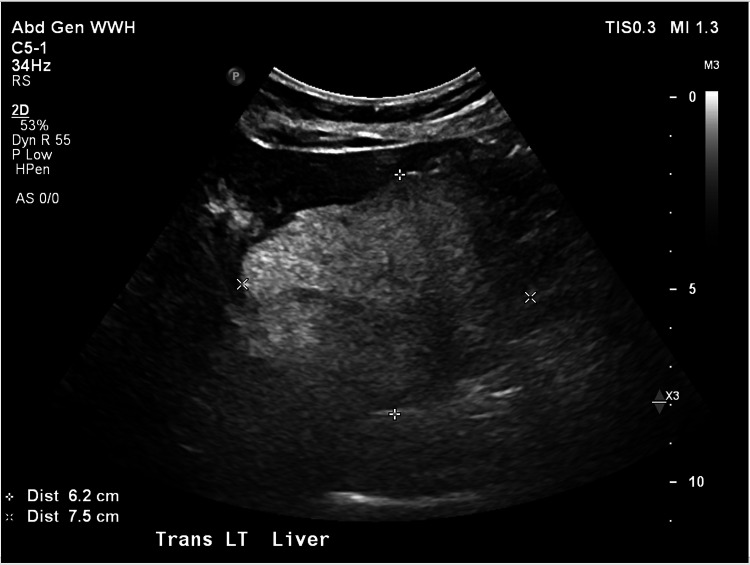
Ultrasound demonstrating a hyperechoic hepatic lesion Transverse grayscale ultrasound image of the left hepatic lobe demonstrates a well-circumscribed hyperechoic mass measuring 6.2 × 7.5 cm (calipers), with mild posterior acoustic attenuation, characteristic of a fat-containing lesion.

Subsequent CT imaging of the abdomen and pelvis, performed two weeks later, demonstrated a 7.0 × 6.5 × 6.3 cm left hepatic lobe mass composed predominantly of macroscopic fat, with intervening enhancing soft-tissue and vascular structures. No biliary ductal dilation or additional hepatic abnormalities were identified (Figure 2).

**Figure 2:**
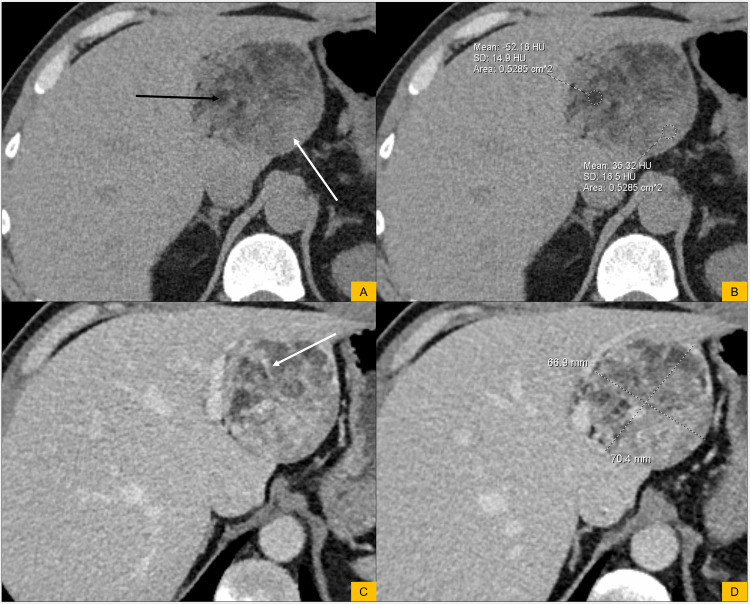
Cross-sectional imaging of a fat-containing left hepatic lobe mass (A, B) Axial non-contrast CT images of the upper abdomen demonstrate a well-circumscribed partially exophytic mass arising from the left hepatic lobe, containing both macroscopic fat (black arrow; mean attenuation −52 Hounsfield units) and soft tissue components (white arrow; mean attenuation 35 Hounsfield units), corresponding to the echogenic lesion identified on ultrasound; (C) Axial contrast-enhanced CT image demonstrates serpiginous enhancing internal structures compatible with intralesional vessels (white arrow); (D) Axial contrast-enhanced CT image demonstrates the lesion measuring up to 7.0 cm in greatest dimension.

Follow-up MRI redemonstrated a heterogeneously enhancing solid mass within the left hepatic lobe containing macroscopic fat, without associated adenopathy or ascites (Figure 3).

**Figure 3:**
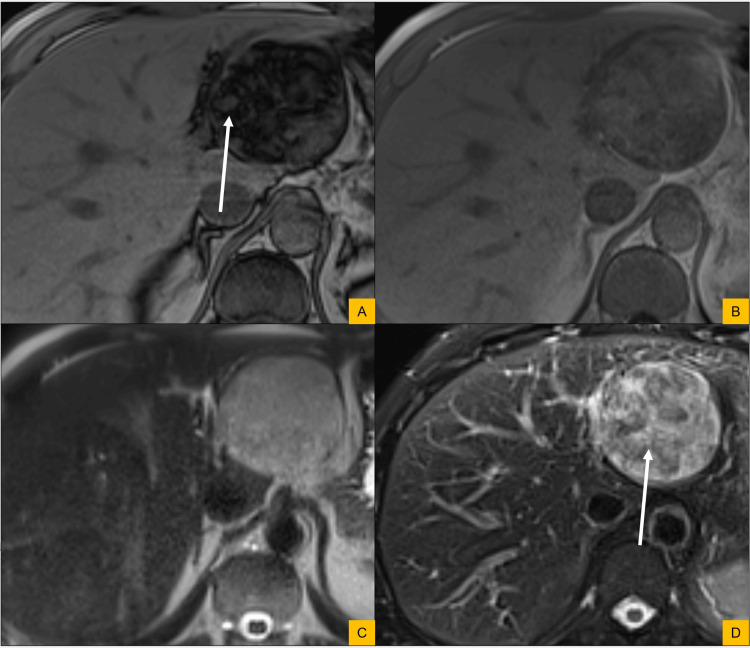
MRI characterization of the fat-containing hepatic mass (A, B) Axial T1-weighted out-of-phase (A) and in-phase (B) MRI images demonstrate hypointense, serpiginous India ink artifact within the lesion on out-of-phase imaging (white arrow), not seen on the corresponding in-phase image, consistent with signal cancellation at macroscopic fat–soft-tissue interfaces; (C, D) Axial T2-weighted non-fat-saturated (C) and fat-saturated (D) images demonstrate signal suppression within the fatty components of the lesion on fat-saturated imaging (white arrow).

Based on the imaging findings, differential considerations included hepatic angiomyolipoma, hepatocellular carcinoma with fatty change, giant hemangioma, pancreatic neoplasm, bowel neoplasm, and mesenteric lipoma. Given the indeterminate nature of the lesion, an ultrasound-guided core biopsy was pursued. Histologic examination demonstrated a neoplastic proliferation composed of mature adipose tissue and eosinophilic epithelioid cells intimately associated with thick-walled blood vessels. No significant cytologic atypia or mitotic activity was identified. Immunohistochemical studies showed focal, patchy positivity for HMB45 and Melan-A, diffuse positivity for GPNMB and SMA; whereas staining for HepPar-1, glypican-3, CAM5.2, CK7, S100, and desmin was negative. The morphologic and immunophenotypic findings supported the diagnosis of angiomyolipoma (Figures 4, 5).

**Figure 4:**
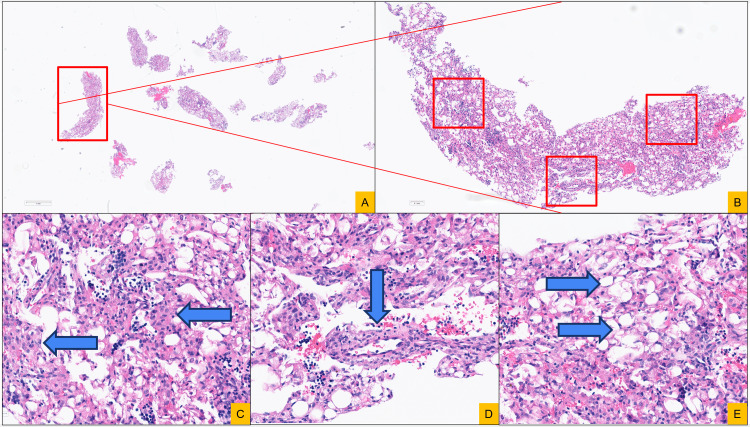
Targeted liver lesion biopsy (A) Low-power view of the liver biopsy specimen demonstrating multiple fragmented cores involved by a mesenchymal neoplasm (H&E, 20×). Background hepatic parenchyma is not represented in the biopsy specimen; (B) Higher-power view of the boxed area in panel A showing an admixture of epithelioid tumor cells, mature adipose tissue, and thick-walled blood vessels (H&E, 100×); (C) High-power view highlighting epithelioid tumor cells with abundant eosinophilic to focally clear cytoplasm (arrows) (H&E, 400×). (D) Thick-walled blood vessels within the lesion (arrow) (H&E, 400×); (E) Mature adipose tissue component within the tumor (arrows) (H&E, 400×).

**Figure 5:**
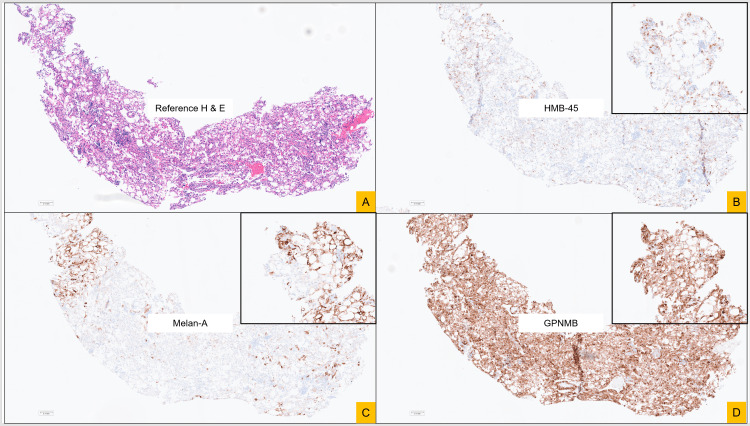
Immunohistochemical characterization of the hepatic lesion. (A) Representative hematoxylin and eosin (H&E)-stained section demonstrating the tumor morphology (100×); (B) Tumor cells show focal patchy cytoplasmic positivity for HMB45 (100×). Inset: higher magnification view (200×); (C) Tumor cells show focal patchy cytoplasmic positivity for Melan-A (100×). Inset: higher magnification view (200×); (D) Tumor cells demonstrate diffuse cytoplasmic expression of GPNMB (100×). Inset: higher magnification view (200×).

Following this diagnosis, the patient underwent an open exploratory laparotomy with lysis of adhesions, intraoperative hepatic ultrasound, and left lateral hepatic sectionectomy (segments II and III). After mobilization of the left lateral hepatic section, intraoperative ultrasound was used to confirm tumor localization, assess its relationship to the left portal vein and hepatic venous structures, exclude vascular invasion, identify the segment II/III portal pedicles, and guide a parenchymal-sparing resection strategy. A left lateral hepatic segmentectomy (segments II and III) was subsequently performed with preservation of the main left portal pedicle and segment IV inflow. Gross examination confirmed complete excision with negative margins. 

The lateral segmentectomy specimen consisted of a 278.2 g segment of liver measuring 14.2 × 10.4 × 8.1 cm. The external capsular surface was intact, smooth, and red-brown. On sectioning, there was a well-defined tan-brown, friable, rubbery, and heterogeneous mass measuring 8.6 × 7.1 × 5.7 cm, associated with focal thinning of the overlying capsule and outward bulging of the capsular surface. The mass abutted the liver capsule and was located 0.3 cm from the parenchymal resection margin. The surrounding uninvolved hepatic parenchyma was tan-brown, smooth, and spongy in appearance (Figure 6).

**Figure 6:**
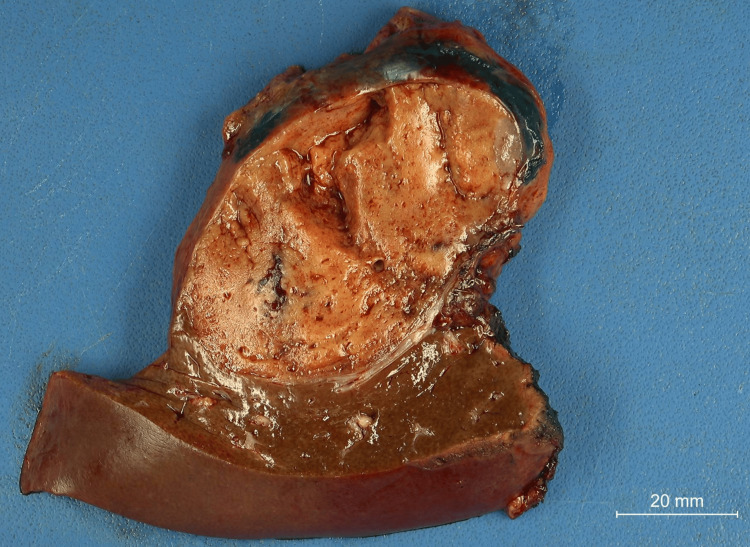
Gross appearance of the hepatic mass The sectioned left lateral segmentectomy specimen demonstrates a well-circumscribed tan-brown, friable, rubbery mass measuring 8.6 × 7.1 × 5.7 cm with a heterogeneous cut surface. The lesion bulges beneath and abuts the hepatic capsule (inked green) and is located 0.3 cm from the parenchymal resection margin (inked black). The surrounding uninvolved hepatic parenchyma is tan-brown and unremarkable.

Microscopic evaluation revealed a well-circumscribed lesion composed of an intimate mixture of thick-walled ectatic vessels, bundles, and nests of epithelioid to spindle smooth muscle-appearing cells with eosinophilic to focally clear cytoplasm and scattered mature adipocytes. The tumor lacked significant cytologic atypia, coagulative necrosis, and increased mitotic activity (Figure 7).

**Figure 7:**
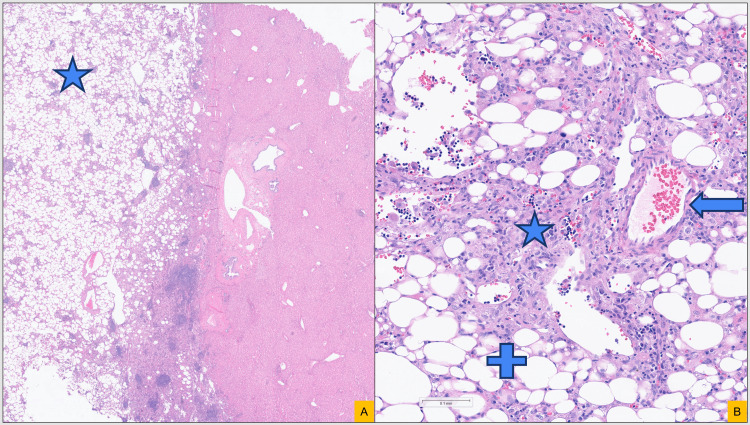
Histologic features of the hepatic resection specimen (A) Low-power view of the resection specimen demonstrating a sharp interface between the angiomyolipoma (star, left) and adjacent non-neoplastic hepatic parenchyma (right) (H&E, 20×); (B) Higher-power view highlighting the characteristic triphasic morphology of the lesion, including thick-walled blood vessels (arrow), epithelioid tumor cells (star), and mature adipose tissue (cross) (H&E, 200×).

Final surgical pathology confirmed angiomyolipoma with negative resection margins, with the closest margin measuring 0.3 cm. Background non-neoplastic hepatic parenchyma was unremarkable. Gross examination confirmed complete excision, and no additional hepatic lesions, carcinomatosis, bile leak, or vascular compromise were identified intraoperatively.

The postoperative course was uncomplicated, and the patient was discharged home on postoperative day three with acetaminophen for pain management. At the most recent follow-up evaluation on postoperative day 20, the patient remained without postoperative complications.

## Discussion

Hepatic angiomyolipoma (HAML) is a rare mesenchymal liver tumor, representing approximately 0.4% of all primary liver tumors in one large institutional series of 5680 cases [7]. It predominantly affects middle-aged women, with most cases reported in patients between the fourth and sixth decades of life. A strong female predominance has been consistently noted across published case series [1].

HAML is classified within the PEComa family, a group of mesenchymal neoplasms characterized by perivascular epithelioid cell differentiation and co-expression of melanocytic and smooth muscle markers. The pathogenesis of HAML is closely linked to the molecular biology of PEComas and to their established association with Tuberous Sclerosis Complex (TSC), an autosomal-dominant disorder caused by mutations in the TSC1 or TSC2 genes [1].

At the molecular level, inactivation of TSC1 or TSC2 leads to constitutive activation of the mechanistic target of rapamycin complex 1 (mTORC1) signaling pathway, which is considered the principal oncogenic driver in many PEComas. Under normal physiologic conditions, the protein products, hamartin (TSC1) and tuberin (TSC2), form a regulatory complex that suppresses the mTORC1 activity. Loss-of-function alterations affecting either gene disrupt this inhibitory pathway, resulting in dysregulated cellular proliferation and growth of the vascular, smooth muscle, and adipocytic components characteristic of HAML [8].

A recent genomic study by Giannikou et al. found no recurrent driver mutations of clinical significance beyond these TSC alterations in HAML [9]. Extra-hepatorenal PEComas frequently harbor TFE3 gene rearrangements as primary oncogenic drivers. While Jimbo et al. demonstrated that, although 21% of HAMLs overexpress the TFE3 protein by immunohistochemistry, FISH analysis showed no corresponding gene arrangements or amplifications [10].

Radiologically, HAML classically presents as a hypervascular, fat-containing solid liver mass with rapid arterial-phase enhancement of the non-fat-containing components. Typical imaging findings include heterogeneously echogenic lesions on ultrasound and low-attenuating lipomatous areas on unenhanced CT. MRI is highly sensitive in detecting even small amounts of adipose tissue through characteristic signal cancellation at the interface between fat-containing and water-containing tissues on opposed-phase sequences, resulting in chemical shift/India ink artifactor through relative hypointensity of fatty tissue on fat-suppressed sequences. Most lesions demonstrate arterial and persistent enhancement without washout. Contrast washout can occur particularly in lesions with abundant vascularity, whereas lesions with fewer vessels tend to show persistent enhancement.

A significant diagnostic challenge arises, particularly with hypervascular lesions with minimal fat component. These lesions exhibiting intense arterial enhancement and subsequent washout on delayed phases closely mimic hepatocellular carcinoma (HCC) or other hypervascular malignancies. Even when fat components are identified, preoperative radiological diagnosis of HAML remains challenging, with reported diagnostic accuracy of only 18-26%. Therefore, in most instances, histopathological and immunohistochemical confirmation is often necessary for a definitive diagnosis [1,4].

AML is histologically characterized by its classic triphasic morphology consisting of a heterogeneous mixture of thick-walled blood vessels, spindle and epithelioid smooth muscle cells, and mature adipose tissue [11]. However, AML demonstrates a broad morphologic spectrum, including component-predominant and other uncommon histologic variants. In some tumors, one component may predominate, resulting in smooth muscle-predominant (fat-poor), lipomatous-predominant, vascular-predominant, or epithelioid morphologies [12]. Because the characteristic triphasic morphology may not be fully represented in limited biopsy specimens, particularly in component-predominant lesions, accurate diagnosis may be challenging and often requires careful immunohistochemical correlation. Among these variants, the epithelioid subtype is of clinical importance because it has been associated with a greater risk of aggressive behavior. Notably, the criteria for defining EAML are more well established in renal AML, where several major series have used a cutoff of >80% epithelioid histology [13-15]. In contrast, hepatic AML more frequently demonstrates epithelioid morphology and monotypic/fat-poor patterns. The threshold for defining hepatic EAML has been less uniformly standardized across studies, with reported cutoffs ranging from as low as 10% epithelioid morphology in earlier reports to predominant (>50%) or even ≥80% epithelioid-cell composition in more recent series [16-18]. In a literature review by Calame et al., the majority of HAML cases exhibiting aggressive clinical behavior were epithelioid. Histologic features associated with more aggressive behavior include large tumor size (>5 cm), high nuclear grade, infiltrative growth, necrosis, and increased mitotic activity (>1/50 high-power field) [1].

Immunohistochemically, HAML exhibits a characteristic profile consistent with its origin in the PEComa family. As previously noted, the hallmark of the PEComa family is the co-expression of melanocytic and smooth muscle markers. Among the melanocytic markers, HMB-45 and Melan-A are the most sensitive and reliable indicators, while microphthalmia transcription factor (MiTF) is also frequently expressed. Among the smooth muscle markers, SMA, desmin, and h-caldesmon are typically positive, reflecting the tumor's smooth muscle differentiation [19,20].

While HMB-45, Melan-A, and SMA are the traditional IHC panel for diagnosing HAML, each marker carries important limitations that can lead to diagnostic uncertainty. Staining intensity and extent of HMB-45 are morphology-dependent. Epithelioid smooth muscle cells show stronger and more widespread HMB-45 reactivity than spindled cells. This means that tumors with a predominantly spindle-cell or lipomatous pattern may show only focal or weak staining, potentially leading to false-negative results [21]. Like HMB-45, Melan-A staining tends to be focal rather than diffuse [22]. In this case, tumor cells only showed rare, patchy cytoplasmic positivity for HMB-45 and Melan-A, highlighting the inconsistency of these markers. SMA is highly sensitive for HAML but is also expressed in a wide range of mesenchymal and non-mesenchymal tumors with smooth muscle components. The lack of specificity of SMA makes it a supportive rather than diagnostic marker [23].

GPNMB has recently emerged as a highly sensitive immunohistochemical biomarker for the PEComa family, including HAML. GPNMB is a lysosomal transmembrane glycoprotein whose expression is regulated by the TSC/mTOR-TFE pathway, making it a biologically relevant marker for these tumors. In a series of 399 mesenchymal tumors, GPNMB was positive in all PEComas, with the majority showing diffuse, moderate-to-strong staining [6]. In another large series of 934 cases, GPNMB was diffusely positive in 94.8% (218/230) of TSC/mTOR-TFE-related neoplasms, including PEComa/AML.5 GPNMB often exhibits diffuse and strong staining even in cases where traditional melanocytic markers, such as HMB-45 or Melan-A, only show focal or equivocal staining. Therefore, as demonstrated in our case, GPNMB serves as an invaluable diagnostic tool, particularly for confirming HAML in challenging cases where conventional immunohistochemical profiles are not definitive.

The differential diagnosis of HAML is broad, as its imaging appearance can mimic several other liver tumors, including hepatocellular carcinoma, hepatic adenoma, hemangioma, and focal nodular hyperplasia, depending on imaging modality. On histology, HAML may also be confused with melanoma, clear cell sarcoma, gastrointestinal stromal tumor, or other mesenchymal tumors, depending on the predominant cell type [1].

Regarding treatment, the decision between surgical resection and observation depends on several factors. Surgical resection is generally recommended when the tumor is symptomatic, larger than 5 cm, shows progression on imaging, or when histological diagnosis remains uncertain after tissue biopsy. For patients who are asymptomatic and have no high-risk features on biopsy, conservative management with regular imaging follow-up is a reasonable option. mTOR inhibitors, such as sirolimus and everolimus, have shown efficacy in reducing tumor volume in patients with AML associated with tuberous sclerosis complex and may be considered an alternative when surgery is not feasible [1].

In terms of prognosis, HAML most commonly follows a benign clinical course, with an overall mortality rate of less than 1%, according to a review article by Klompenhouwer et al. [1,24]. However, aggressive behavior, including local recurrence and distant metastasis, has been reported, particularly in tumors with epithelioid histology, large size (> 5 cm), cytological atypia, necrosis, and high mitotic activity. Because these features are difficult to determine pre-resection, long-term radiological follow-up is recommended even after resection, with the first evaluation suggested at one year and subsequent monitoring based on tumor behavior.

In summary, this case highlights several important diagnostic challenges associated with hepatic angiomyolipoma, a rare PEComa-family neoplasm with highly variable radiologic, morphologic, and immunophenotypic features. The broad histologic spectrum of HAML, including component-predominant and other uncommon variants, may obscure the classic triphasic morphology, particularly in limited biopsy specimens, and complicate distinction from other hypervascular hepatic neoplasms. Although conventional melanocytic markers such as HMB45 and Melan-A remain central to the diagnosis of HAML, their expression may be focal or limited in some tumors, potentially creating diagnostic uncertainty. In the present case, diffuse GPNMB expression provided a particularly valuable diagnostic clue and strongly supported classification as a PEComa despite only focal expression of conventional melanocytic markers. As recognition of the morphologic diversity of HAML continues to expand, GPNMB may serve as a useful ancillary immunohistochemical marker in diagnostically challenging HAMLs and other PEComa-family neoplasms.

## Conclusions

Hepatic angiomyolipoma is a rare PEComa-family neoplasm that may pose significant diagnostic challenges due to its broad morphologic spectrum and variable radiologic and immunophenotypic features. Component-predominant and other uncommon histologic variants may obscure the classic triphasic morphology, particularly in limited biopsy specimens, and complicate distinction from other hypervascular hepatic neoplasms. This case highlights the potential diagnostic utility of GPNMB as an ancillary immunohistochemical marker in challenging HAMLs that demonstrate only focal expression of conventional melanocytic markers, such as HMB45 and Melan-A. Increased recognition of the morphologic diversity of HAML and the supportive role of GPNMB may improve diagnostic accuracy in difficult cases.

## References

[1] Calame P, Tyrode G, Weil Verhoeven D (2021). Clinical characteristics and outcomes of patients with hepatic angiomyolipoma: a literature review. World J Gastroenterol.

[2] Izubuchi Y, Tanaka T (2025). PEComa-its clinical features, histopathology, and current therapy. Jpn J Clin Oncol.

[3] Elsayes KM, Narra VR, Lewis JS, Jr. Jr., Brown JJ (2005). Magnetic resonance imaging of adrenal angiomyolipoma. J Comput Assist Tomogr.

[4] Seow J, McGill M, Wang W, Smith P, Goodwin M (2020). Imaging hepatic angiomyolipomas: key features and avoiding errors. Clin Radiol.

[5] Li H, Matoso A (2025). Unlocking diagnostic potential: A retrospective analysis of GPNMB immunohistochemistry in nearly 1000 surgical pathology specimens. Hum Pathol.

[6] Wangsiricharoen S, Ingram DR, Morey RR, Wani K, Lazar AJ, Wang WL (2024). Glycoprotein nonmetastatic melanoma protein B (GPNMB) immunohistochemistry can be a useful ancillary tool to identify perivascular epithelioid cell tumor. Mod Pathol.

[7] Jung DH, Hwang S, Hong SM (2018). Clinico-pathological correlation of hepatic angiomyolipoma: a series of 23 resection cases. ANZ J Surg.

[8] Bissler JJ, Kingswood JC, Radzikowska E (2013). Everolimus for angiomyolipoma associated with tuberous sclerosis complex or sporadic lymphangioleiomyomatosis (EXIST- 2): a multicentre, randomised, double-blind, placebo-controlled trial. Lancet.

[9] Giannikou K, Klonowska K, Tsuji J (2023). TSC2 inactivation, low mutation burden and high macrophage infiltration characterise hepatic angiomyolipomas. Histopathology.

[10] Jimbo N, Nishigami T, Noguchi M (2017). Hepatic angiomyolipomas may overexpress TFE3, but have no relevant genetic alterations. Hum Pathol.

[11] Calio A, Brunelli M, Segala D (2021). Angiomyolipoma of the kidney: from simple hamartoma to complex tumour. Pathology.

[12] Nonomura A, Enomoto Y, Takeda M, Takano M, Morita K, Kasai T (2012). Angiomyolipoma of the liver: a reappraisal of morphological features and delineation of new characteristic histological features from the clinicopathological findings of 55 tumours in 47 patients. Histopathology.

[13] He W, Cheville JC, Sadow PM (2013). Epithelioid angiomyolipoma of the kidney: pathological features and clinical outcome in a series of consecutively resected tumors. Mod Pathol.

[14] Nese N, Martignoni G, Fletcher CD (2011). Pure epithelioid PEComas (so-called epithelioid angiomyolipoma) of the kidney: a clinicopathologic study of 41 cases: detailed assessment of morphology and risk stratification. Am J Surg Pathol.

[15] Brimo F, Robinson B, Guo C, Zhou M, Latour M, Epstein JI (2010). Renal epithelioid angiomyolipoma with atypia: a series of 40 cases with emphasis on clinicopathologic prognostic indicators of malignancy. Am J Surg Pathol.

[16] Dalle I, Sciot R, de Vos R (2000). Malignant angiomyolipoma of the liver: a hitherto unreported variant. Histopathology.

[17] Yan Z, Grenert JP, Joseph NM, Ren C, Chen X, Shafizadeh N, Kakar S (2018). Hepatic angiomyolipoma: mutation analysis and immunohistochemical pitfalls in diagnosis. Histopathology.

[18] Yoo Y, Kim J, Song IH (2025). Risk prediction criteria for the primary hepatic perivascular epithelioid cell tumour family, including angiomyolipoma: analysis of 132 cases with a literature review. Histopathology.

[19] Zavala-Pompa A, Folpe AL, Jimenez RE (2001). Immunohistochemical study of microphthalmia transcription factor and tyrosinase in angiomyolipoma of the kidney, renal cell carcinoma, and renal and retroperitoneal sarcomas: comparative evaluation with traditional diagnostic markers. Am J Surg Pathol.

[20] Schoolmeester JK, Howitt BE, Hirsch MS, Dal Cin P, Quade BJ, Nucci MR (2014). Perivascular epithelioid cell neoplasm (PEComa) of the gynecologic tract: clinicopathologic and immunohistochemical characterization of 16 cases. Am J Surg Pathol.

[21] Makhlouf HR, Ishak KG, Shekar R, Sesterhenn IA, Young DY, Fanburg-Smith JC (2002). Melanoma markers in angiomyolipoma of the liver and kidney: a comparative study. Arch Pathol Lab Med.

[22] Roma AA, Magi-Galluzzi C, Zhou M (2007). Differential expression of melanocytic markers in myoid, lipomatous, and vascular components of renal angiomyolipomas. Arch Pathol Lab Med.

[23] Miettinen M (2014). Smooth muscle tumors of soft tissue and non-uterine viscera: biology and prognosis. Mod Pathol.

[24] Klompenhouwer AJ, Verver D, Janki S (2017). Management of hepatic angiomyolipoma: a systematic review. Liver Int.

